# Enhancing supercapacitor performance through design optimization of laser-induced graphene and MWCNT coatings for flexible and portable energy storage

**DOI:** 10.1038/s41598-023-48518-2

**Published:** 2023-11-30

**Authors:** Hassan Tariq, Saif Ullah Awan, Danish Hussain, Syed Rizwan, Saqlain A. Shah, Sana Zainab, M. Bilal Riaz

**Affiliations:** 1 Department of Electrical Engineering, College of Electrical and Mechanical Engineering, National University of Sciences and Technology (NUST), Islamabad, 44000 Pakistan; 2 Department of Mechatronics Engineering, NUST College of Electrical and Mechanical Engineering, National University of Sciences and Technology (NUST), Islamabad, 44000 Pakistan; 3 Physics Characterization and Simulation Lab (PCSL), Department of Physics, School of Natural Sciences (SNS), National University of Sciences and Technology (NUST), Islamabad, 44000 Pakistan; 4 Department of Physics, Forman Christian College (University), Lahore, Pakistan

**Keywords:** Chemical engineering, Electrochemistry, Energy, Environmental chemistry, Green chemistry, Materials chemistry, Surface chemistry, Energy infrastructure, Energy storage, Condensed-matter physics, Materials for energy and catalysis, Nanoscale materials, Soft materials, Graphene, Nanoscale devices, Techniques and instrumentation, Applied physics, Chemical physics, Condensed-matter physics, Electronics, photonics and device physics, Quantum physics

## Abstract

The field of supercapacitors consistently focuses on research and challenges to improve energy efficiency, capacitance, flexibility, and stability. Low-cost laser-induced graphene (LIG) offers a promising alternative to commercially available graphene for next-generation wearable and portable devices, thanks to its remarkable specific surface area, excellent mechanical flexibility, and exceptional electrical properties. We report on the development of LIG-based flexible supercapacitors with optimized geometries, which demonstrate high capacitance and energy density while maintaining flexibility and stability. Three-dimensional porous graphene films were synthesized, and devices with optimized parameters were fabricated and tested. One type of device utilized LIG, while two other types were fabricated on LIG by coating multi-walled carbon nanotubes (MWCNT) at varying concentrations. Characterization techniques, including scanning electron microscopy (SEM), atomic force microscopy (AFM), X-ray diffraction (XRD), Raman spectroscopy, and voltammetry, were employed to analyze the fabricated devices. AFM analysis revealed a surface roughness of 2.03 µm for LIG due to laser treatment. SEM images displayed compact, dense, and porous surface morphology. XRD analysis confirmed the presence of graphene and graphene oxide, which was further supported by energy-dispersive X-ray spectroscopy (EDX) data. Raman spectroscopy indicated that the fabricated samples exhibited distinct D and G bands at 1362 cm^–1^ and 1579 cm^–1^, respectively. Cyclic voltammetry (CV) results showed that LIG's capacitance, power density, and energy density were 6.09 mF cm^–2^, 0.199 mW cm^–2^, and 3.38 µWh cm^–2^, respectively, at a current density of 0.2 mA cm^–2^. The LIG-MWCNT coated electrode exhibited a higher energy density of 6.05 µWh cm^–2^ and an areal-specific capacitance of 51.975 mF cm^–2^ compared to the LIG-based devices. The fabricated device has potential applications in smart electronics, nanorobotics, microelectromechanical systems (MEMS), and wearable and portable electronics.

## Introduction

The increasing demand for efficient, portable, and eco-friendly energy storage solutions is driving the development of supercapacitors and batteries with high energy and power densities. These energy storage technologies have a wide range of applications, from miniature devices to large electric vehicles and grid-scale energy storage systems, generating significant interest in their advancement and implementation^1^. Supercapacitors (SCs) bridge the gap between capacitors and batteries by offering higher power densities (rapid power delivery) and higher energy densities (power storage capacity) than conventional capacitors^2^. These attributes make supercapacitors an appealing option in our fast-paced world, where energy storage solutions that provide high power and quick charging capabilities are essential. SCs show great potential as energy storage devices that could complement or even replace lithium-ion batteries in wearable and stretchable microelectronics. However, SCs exhibit relatively low energy density and limited mechanical stretchability^3,4^. In contrast, they also benefit from a large surface area, high power density, low energy density, rapid charging rate, and excellent cycle stability^5^. Critical factors in the charge storage process of double-layer SCs include the electrode/electrolyte junction, electrical conductivity, average pore diameter, and electrode surface volume^6–8^. Nevertheless, structural degradation and inadequate interfacing between the electrode and current collector can compromise the electrical performance of these devices^9^.

To maintain a leading role in the world of multifunctional and flexible energy storage technologies, SCs require increased power and energy densities. Consequently, developing new electrode formulations that deliver higher capacitive performances without fatigue failure is crucial. Recent advances and challenges in creating nanostructured and nano-engineered materials have emphasized the need for energy storage devices with mechanical robustness, multifunctional resilience, adaptability, and integration to enable more attractive, lightweight, compact, and intelligent designs^10–13^. Electrochemical energy storage is now vital for various applications, including portable medical and electrical devices, as well as ground and aerial vehicles. Conventional supercapacitors and batteries often cannot be easily integrated into emerging technologies such as smart textiles, electronic magazines, e-books, packages with data-collection capabilities, flexible wearable electronics and displays, flexible solar cells, epidermal sensors, and others. This is due to design limitations concerning aesthetics, convenience, system simplicity, and reliability, which impede their seamless integration with these new applications. Conventional energy storage technologies are often limited in functionality, designed for a single purpose, and unable to adapt to different geometries. Moreover, they do not offer additional features such as load-bearing or impact/ballistic protection, which could reduce the overall weight or volume of the system. As flexible electronic devices become more affordable and the demand for smarter, elastically deformable products increases, energy storage solutions with similar mechanical properties will ensure seamless integration and self-sufficiency^14,15^.

Carbon-based electrodes are popular for supercapacitors due to their large surface area, high conductivity, low contact resitance and increased porosity^16,17^. To enhance the capacitive capabilities of carbon-based electrodes, various adjustments have been made to their morphology, including the creation of layered, porous, 1D, 2D, and 3D nanostructures, among other configurations^18,19^. Supercapacitors made with graphene have several advantages, but graphene production is difficult and expensive due to the multiple chemical stages involved and low yield^20,21^. Other methods, such as direct laser writing on graphite oxide^22–24^, light-scribed DVD drives^25^, and CO_2_ laser-based processes, have also been used to create supercapacitors^26^. Laser-processed graphene-based supercapacitors outperform conventional supercapacitors in terms of volumetric energy performance. A laser machine can shape electrode arrays and reduce the electro-sprayed GO thin layer into laser-processed graphene (LPG) by adjusting the output laser power^27^. A more efficient and affordable method for creating porous graphene with embedded nanoparticles is direct laser scribing, which induces the in situ production of nanoparticles embedded in porous graphene on polyimide films containing metal complexes^28^.

Previously, waste mass was commonly used as a binder and active carbon with carbon-based electrode materials in a current collector; however, this approach reduces capacitance. Binder-free electrode formulations have gained popularity in recent years because they can be used as electrodes immediately. Other chemistries studied for binder-free electrodes include carbon nanotube (CNT) sponge, carbon composite sheets, and carbon cloth, among others. However, these components must be combined with the current collector to assemble the device, which once again impedes efficient charge transfer from the electrode material to the active material and reduces the capacitive charge storage capacity^29^. Nevertheless, various morphologies can be designed to improve electrode/electrolyte interaction and surface charge storage. CNT networks are highly durable and flexible, making them ideal for wearable electronics, as they can withstand twisting, scouring, and stretching without impacting their functionality^30–35^. A potential method for enhancing charge storage is employing novel electrode chemistries that allow for partial in-situ surface conversion. The enhanced charge storage of the modified surface will be reinforced by the strong connection between the active surface and the intact substrate, enabling efficient charge transfer and maximizing capacitance. Pulsed nanomaterials via laser carbonization show potential as supercapacitor electrodes. The capacitance of these electrodes can be influenced by various factors, such as the type of electrolyte used, the substrate material, surface area, and laser scanning speed^36–38^.

In this study, we use a CO_2_ laser to synthesize laser-induced graphene (LIG) in a single step at a low cost. We investigate the coating of MWCNTs on LIG to fabricate flexible electrodes and study their electrical properties (in terms of power density, energy density, and capacitance) for supercapacitors. To modify the polyimide's surface, we employ a simple laser setup. Polyimide is an insulating and non-conductive polymer; however, when exposed to laser light, its surface transforms into a highly porous carbonized material known as laser-induced graphene (LIG). This LIG exhibits significantly higher conductivity compared to a standard polyimide sheet. Due to the high cost and difficulty in obtaining graphene, we aimed to fabricate an electrode with similar structural properties to graphene and graphene oxide. The high density and porous structure of the manufactured LIG make it well-suited for supercapacitor applications. In addition, MWCNTs are coated to enhance LIG's stability and flexibility without compromising the electrical performance of the device. The coating of the LIG electrode with MWCNT enhances the device's conductivity, flexibility, and electrical performance due to its highly porous structure, resulting in a higher capacitance of 11.17 mF cm^−2^ and a higher energy density of 6.05 µF cm^−2^.

## Materials and methods

### Laser scribing technique

We used commercial polyimide sheets (200 µm thickness) as a substrate for fabricating flexible supercapacitor (FSC) electrodes due to their excellent thermal stability, mechanical strength, and chemical resistance. A laser-scribing process was employed to reduce the polyimide into conductive laser-induced graphene (LIG) electrodes using a low-frequency (405 nm) light source for in-situ transformation. As laser power increased, the oxidation process in the polyimide material accelerated, resulting in significant graphitization of the LIG material, which is crucial for conductivity. A CO_2_ laser was used to etch the interdigitated electrodes (IDE) on a polyimide substrate. Cike 8.2 software helped control the laser and adjust settings like programming speed, linearity, and rapidity to achieve the desired results. The optimal laser power for the device design was determined to be 80 mm/min with 0.5 linearity and 0.36 rapidity.

### Designing and fabrication of electrode by laser scribing technique

**Figure 1:**
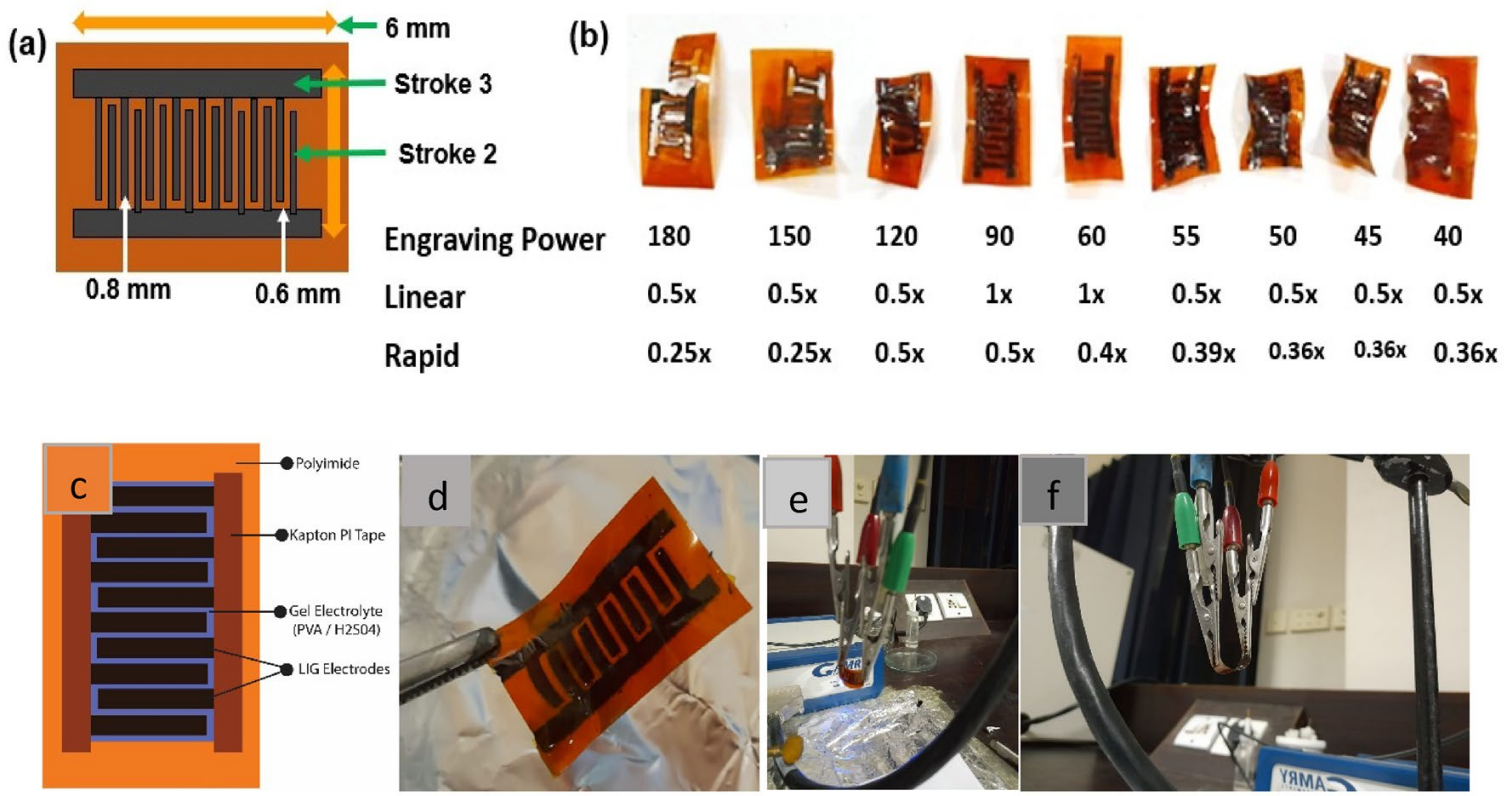
(**a**) Schematic diagram of interdigitaed comb like electrode using Laser machine with design parameters, (**b**) design optimization using different Laser powers, (**c**) LIG (LASER induded Graphene) electrode Design using Adobe Illustrator 20, (**d**) displays a picture of PI (polyimide) and LIG materials that exemplifies how laser irradiation has altered the surface of PI material, (**e**) the manufactured electrode was bent over to check the stability of the device, (**f**) flexible device that was bent at angle 45° for CV measurements was clipped by crocodile switches.

**Figure 2:**
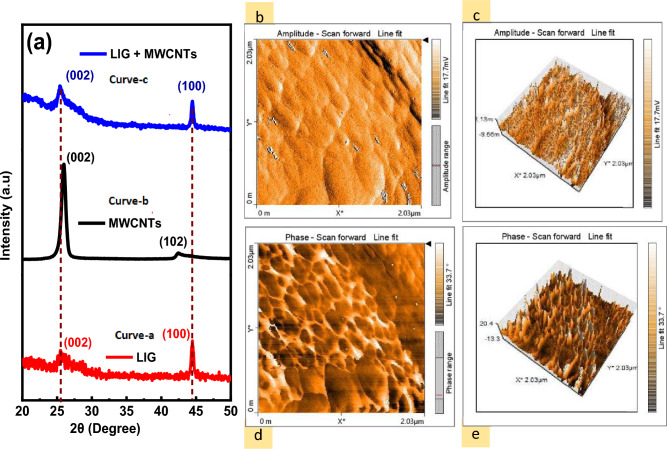
(**a**) Red color curve represents LIG exhibits an XRD pattern that is composed of diffraction from the (002) and (100) planes, the black color curve represents MWCNT X-ray diffraction pattern composed of diffraction from the (002) and (102) planes, the blue color curve represents X-ray diffraction pattern of (LIG coated with MWCNTs), (**b**) 2D AFM scan of a Polyimide, (**c**) 3D AFM scan of a Polyimide, (**d**) 2D AFM image scan of Laser treated LIG, (**e**) 3D AFM scan of Laser treated LIG.

Pure LIG electrodes, as well as 2% and 5% multi-wall carbon nanotubes (CNTs) coated LIG electrodes (referred to as LIG/2%CNTs and LIG/5%CNTs), were fabricated using the laser scribing technique on a 1 cm^2^ polyimide flexible substrate. Design parameters and the fabrication process are illustrated in Figs. 1 and 2. Each interdigitated electrode has a 0.71 mm width and 6 mm length, with a collector width of 1.05 mm and 0.6 mm inter-electrode spacing. The optimization of design variables is crucial for the desired device performance. Cyclic voltammetry testing evaluates the distortion produced in the device. It is observed that if the space between the two parallel interdigitated electrodes is less than 0.6 mm, the island-covered carbon will be closer to the adjacent electrode and might link with it, thereby affecting the efficiency of the device. Furthermore, any deviation from the optimal horizontal spacing of 0.6 mm between the comb-like structures would negatively impact the efficiency of the device.

To increase the surface area of the device and promote ion diffusion, the inter-electrode spacing is kept smaller than the collector width. Specifically, the comb-shaped electrode has width of 0.71 mm, while collector has 1.05 mm width. However, increasing the space between interdigitated electrodes beyond 0.75 mm may result in decreased capacitance and overall device performance. Therefore, the optimization of the design variables is critical to achieve the desired device performance. The overall schematic of the electrodes is shown in Fig. 1a. Figure 1b presents design optimization using different Laser powers Fig. 1c showed LIG electrode Design using Adobe Illustrator 20 while Fig. 1d displays a picture of PI (polyimide) and LIG materials that exemplifies how laser irradiation has altered the surface of PI material.

The fabrication process includes the following steps: (1) The interdigitated electrodes were engraved on the PI with an engraving speed and power of 50 mm/min and 0.5 W using a 405 nm semiconductor laser. The resultant LIG material has a substantial surface area, enhanced porosity, and good electrical conductivity. The fabricated electrode exhibits conductive properties between graphene and graphene oxide^36^. (2) The Kapton PI tape was applied to the PI evenly and gently cleaned. (3) A polyvinyl alcohol PVA/H_2_SO_4_ gel electrolyte was applied to the active regions to guarantee its diffusion into the electrodes during overnight drying. The PVA/H_2_SO_4_ gel electrolyte was prepared using a standard protocol^26^. Firstly, 1 g of PVA was dissolved in 8 ml of deionized water and sonicated for 30 min at room temperature. Then, 1.2 ml of H_2_SO_4_ was added to the solution, and the resultant mixture was allowed to settle for 24 h. To fabricate MWCNTs coated LIG electrode, CTAB (0.25 mg) was dispersed in 300 µL deionized water, followed by adding 2 mg or 5 mg of MWCNTs to the solution. The resulting mixture was sonicated for 30 min to ensure proper homogenization. The suspension was spray-coated onto the LIG film and heated at 35–40 °C using a hotplate. The sample with sprayed MWCNTs was used as SC electrode without further treatment. Steps (2) and (3) were repeated for working of MWCNTs electrodes. Figure 1e shows the mechanical flexibility and stability of the device after bending. The MWCNTs coated flexible device was bent at higher and greater angles to test its flexibility and stability, as seen in Fig. 1f.

### Characterization techniques

X-ray diffraction (XRD) measurements were conducted using a D8 Discover Diffractometer with Cu Kα as the X-ray source. Scanning electron microscopy (SEM) and Energy Dispersive X-ray Spectroscopy were performed for morphological investigation. The electrode's surface roughness was measured via atomic force microscopy (AFM) analysis using a Nanosurf FlexAFM and C3000 controller. AFM was used for the surface characterization of PI and LIG. Tapping mode at a lower cantilever oscillation amplitude of up to 1 nm was used to operate the AFM, as it causes less damage to soft samples. Raman spectra were obtained using a Thermo Renishaw with a λ = 532 nm He–Ne laser. Electrochemical testing was conducted via cyclic voltammetry and Galvanostatic charge/discharge measurements using a Gamry 1010B Potentiostat workstation. The CV voltammograms were recorded in the voltage range of − 1 to 1 V with scan rates between 1 and 200 mVs^−1^. Electrochemical Impedance Spectroscopy (EIS) was acquired at the open circuit potential (OCP) in a frequency range of 20 kHz–10 MHz using a sinusoidal signal of 10 mV.

## Results and discussion

The X-Ray diffraction (XRD) patterns of LIG obtained from commercial polyimide (PI) sheets is revealed in Fig. 2a. Curve (a) displays a prominent peak centered at 2θ = 25.44°, revealing LIG-like structures with significant degrees of graphitization, indicating an interlayer spacing of ~ 3.4 Å between the (002) layers. The increasing interlayer gap is attributed to defects formed on hexagonal graphene layers. Another distinguishing feature of the XRD pattern is the low-intensity peak at 2θ = 44°, which is associated with the reduction peak of PI into graphene-like LIG material. This peak is related to the in-plane structure of LIG and is a reflection from (100) planes. The laser-induced graphitization phenomenon may be caused by the presence of repeated aromatic and amide units in PI, as suggested by few previous studies^18,39–41^. These units are responsible for the formation of graphene-like structures in the LIG material. Curve (b) represents the XRD pattern of a multi-walled carbon nanotube (MWCNT). The strongest and sharpest diffraction peak for MWCNT appears at 2θ = 25.7°, which is labeled as the (002) plane. This peak exhibits a general decline in intensity compared to conventional graphite, 2θ = 26.5°, suggesting an increase in the *sp*^2^, C=C layer distance^42^. Besides, there is also a small diffraction at 2θ = 43.0° for MWCNT. Curve (c) depicts the XRD pattern of multiwall carbon nanotube-coated laser-induced graphene. At 2θ = 25.4°, a modest increase in the peak labeled as 002 indicates an increase in the content of graphene-like structures with high levels of graphitization due to the coating of MWCNTs. The 002 peak is a characteristic peak of graphitic materials and is associated with the stacking of graphene layers along the c-axis. Therefore, the presence of this peak in LIG-MWCNT confirms the formation of graphene-like layered structures. Additionally, the low-intensity peak at 2θ = 44° represents the signature of the reduction peak of PI into graphene-like LIG-MWCNT material. This peak is a reflection from (100). This peak further affirms the reduction of PI into graphene-like LIG-MWCNT material. Equation (1) was used to calculate the crystalline size along the c-axis (Lc).

1 $$ L_{c} = \frac{{0.9{ }\lambda }}{{B_{1/2} \left( {2\theta } \right)\left( {cos\theta } \right)}} $$

where ${B}_{1/2}$(radians) is the FWHM for peak (002) and λ = 1.54 A^°^ (Cu kα source of XRD). L_c_ for LIG and LIG-2mg MWCNT coated is 24 nm and 21 nm, respectively^43,44^_ENREF_26. Overall, the XRD pattern of LIG-MWCNT confirms the successful formation of graphene-like structures and the coating of MWCNTs onto the LIG, which can have potential applications in various fields, such as energy storage, sensing, and catalysis.

Figure 2b,c show a 2D AFM scan of the PI and LIG, respectively, whereas Fig. 2d,e show a 3D AFM scan. The analysis revealed that the PI film had a more compact structure and a smoother surface than LIG. On the other hand, LIG had a surface dominated by islands, and an overall increase in surface roughness is attributed to the laser treatment and subsequent polymer-to-graphitic carbon conversion^43^. The intensity of spikes on the surface increased after photoexcitation. The rough, graphene-like surface of LIG was beneficial for developing a double layer and the adherence of the electrolyte, which improved the capacitive performance of the device.

**Figure 3:**
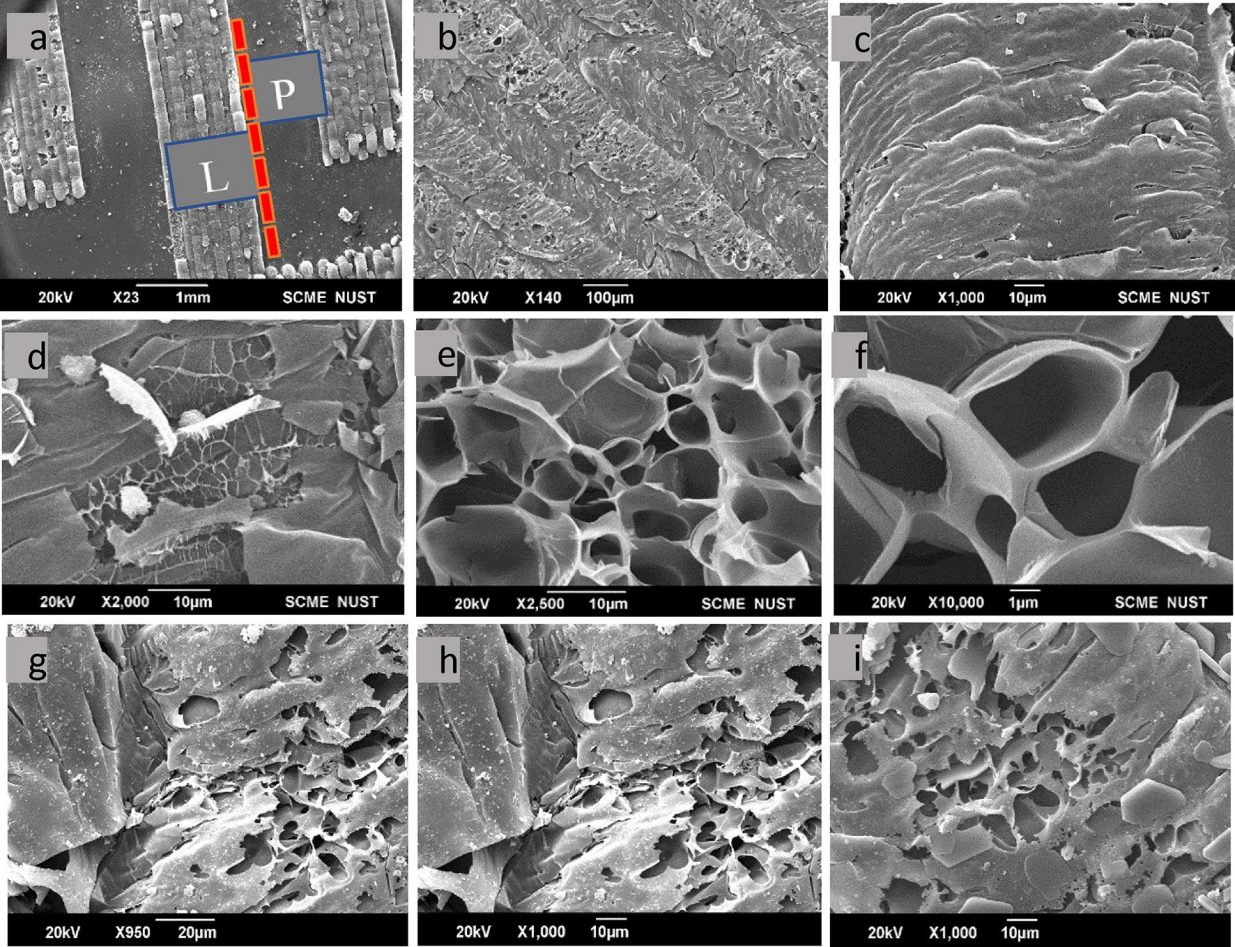
(**a**) SEM micrograph of laser-treated PI into interdigitated electrode at speed of 80 mm/min. The non-treated PI is represented by the region to the right of the red dashed lines, while the LIG region is represented by the region to the left of the dotted line, (**b**) irradiated region represents the LIG material on top of the PI, (**c**) Magnified SEM image of laser treated polyimide sheet, (**d**) side view: SEM image of laser treated polyimide coated with MWCNTs depicted flakes, (**e**) top view: Low resolution SEM image of LIG coated with MWCNTs SEM image showing the carbon flakes; scale bar, 10 µm, (**f**) top view: Higher magnification SEM image of LIG coated with MWCNTs; scale bar, 1 µm resolution, (**g–i**) SEM image of laser treated polyimide coated with MWCNTs showing the porous morphology at different scale bars.

In Fig. 3a, the red dotted line in the diagram represents the non-radiated area of the PI material, while the area beyond the red dashed line corresponds to the LIG region. This figure illustrates the effect of the laser beam on the PI sheet. The laser beam may be causes the termination of volatile C–N, C–O–C, and C=O bonds in the processed region of the PI sheet. This results in changes in the composition and shape of the material, which is reflected by the higher carbon content in the processed PI sheet compared to the unprocessed version^45^. It is possible that some nitrogen and oxygen atoms could be liberated as gases due to the laser-induced photo-thermal activity^46^. Figure 3a illustrates that the resultant content is transformed into porous carbon material. The irradiated region represents the LIG material on top of the PI sheet, as shown in Fig. 3b. This rough surface helps the electrolyte adhere well, which promotes diffusion and ultimately improves capacitance. Figure 3c,d show the magnified SEM image of the laser-ablated polyimide sheet. Figure 3e is an SEM image of the laser-carved merged area of LIG material coated with MWCNTs (multi-walled carbon nanotubes), which demonstrates strong links between the carbon flakes in the material and the meso-micropores on the surface. The Fig. 3f is an SEM image at a different resolution, which shows the porous morphology of the LIG material after it has been coated with MWCNTs. Figure 3g–i are SEM images of LIG coated with MWCNTs. The results depict the formation of carbon flakes through the coating of MWCNTs, which are then organized in an aromatic graphitic structure to provide graphitic content^47^. The resulting honeycomb patterns offer a larger surface area and strong adhesion, making it highly beneficial for energy storage. The stacked flakes create high porosity, and their functional surfaces allow for the diffusion of electrolytes over the active material surface. The stacked flakes create high porosity and offer functional surfaces for the electrolyte diffusion over the active material surface. Creating laser-induced PI or laser-induced graphene involves a photo-thermal process that results in the formation of LIG porous layered flakes. These flakes further enhance the assisted diffusion process after the integration of MWCNTs. Overall, combining MWCNTs coating and laser-induced PI/LIG results in a highly beneficial material for energy storage applications, with enhanced surface area, porosity, and assisted diffusion capabilities.

**Figure 4:**
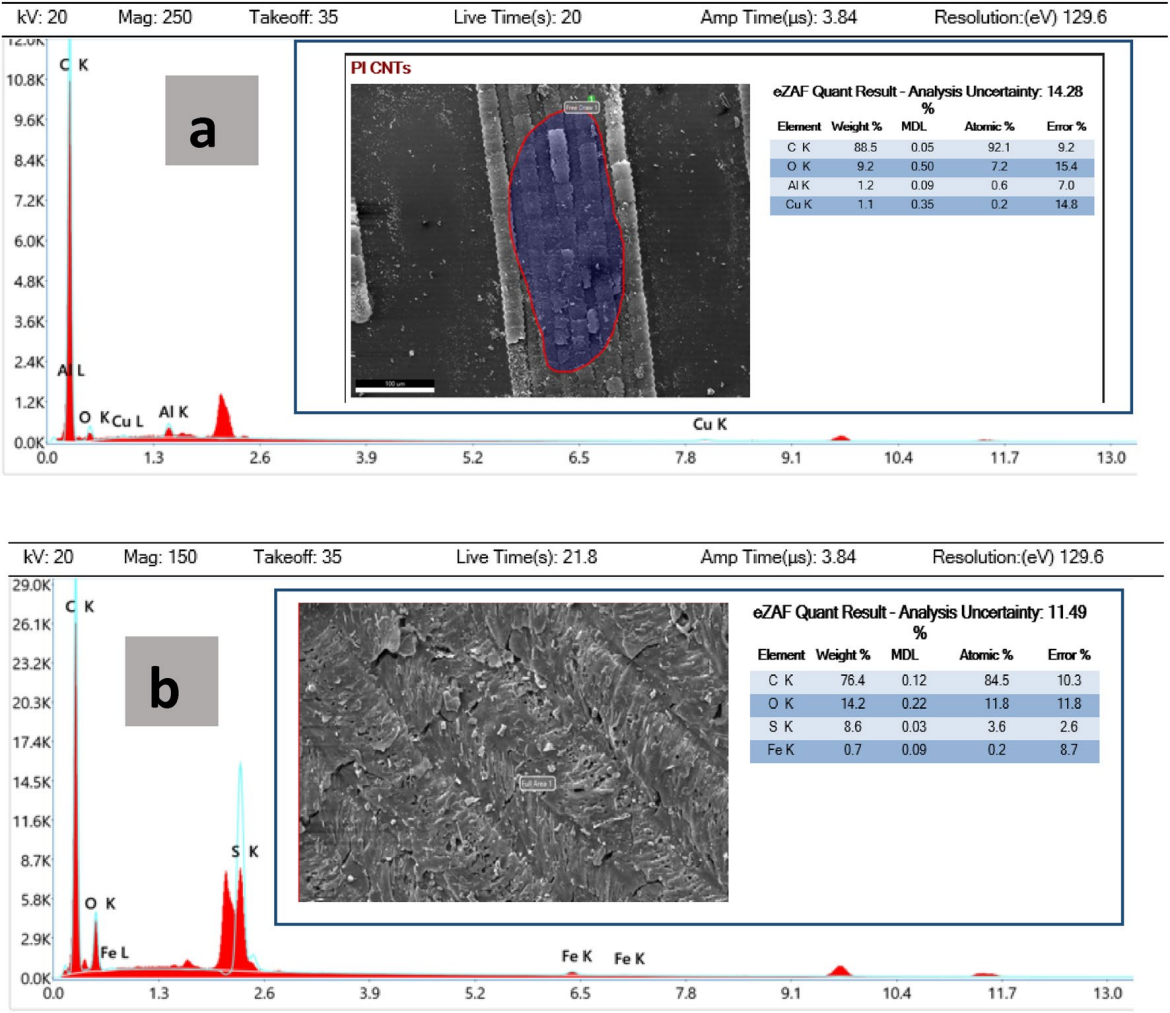
(**a**) Energy dispersive X-ray (EDX) analysis of laser treated PI. Inset (**a**) red marked area shows the presence of various elements in wt% and at.% as depicted in the table. (**b**) Energy dispersive X-ray (EDX) analysis of Laser Induced Graphene coated with MWCNTs (LIG-MWCNTs). Insets showed the presence of various elements in wt% and at.%. Inset (**b**) white marked area shows the presence of various elements in wt% and at.% as depicted in the table.

In Energy Dispersive X-ray Spectroscopy (EDX), an electron beam strikes a sample, causing the ejection of inner shell electrons. An electron from an outer shell fills the resulting vacancy in the inner shell, and this transition releases energy in the form of an X-ray. By detecting the energy and intensity of these X-rays, it is possible to determine the elements present in the sample. Figure 4a,b show the elemental analysis of the samples with and without the coating of MWCNTs, respectively. In laser-induced graphene (LIG), when the PI is reduced, it increases carbon content, which is evident in the LIG's dispersive energy X-ray (EDX) study, showing the highest carbon content. MWCNTs in the sample can also contribute to the increasing carbon content observed in the coated LIG sample. The size of the polymeric stub used in EDX to hold the substrate material is responsible for other visible aspects. The EDX analysis can detect other elements present in the sample. However, the element with the highest concentration typically indicates the target element that was successfully converted.

The EDX data presented in Fig. 4a,b directly reflects the output from our analytical equipment without any selective removal of minority elements. The presence of these elements can be attributed to various sources within our experimental setup. Iron (Fe) and Copper (Cu) may originate from the commercial-grade polyimide sheets and multi-walled carbon nanotubes (MWCNTs) used in our research, as trace amounts of these elements can be present in these source materials. Sulfur (S) is attributed to the use of polyvinyl alcohol (PVA) and sulfuric acid (H_2_SO_4_) in our gel electrolyte, as the composition of H_2_SO_4_ inherently contains sulfur. Additionally, the introduction of Aluminum (Al) in our samples can be linked to the use of Al-foil during sample handling and placement in desiccators, with the potential for Al contamination during this process. These observations provide valuable insights into the origin of the elements detected in the EDX analysis.

**Figure 5:**
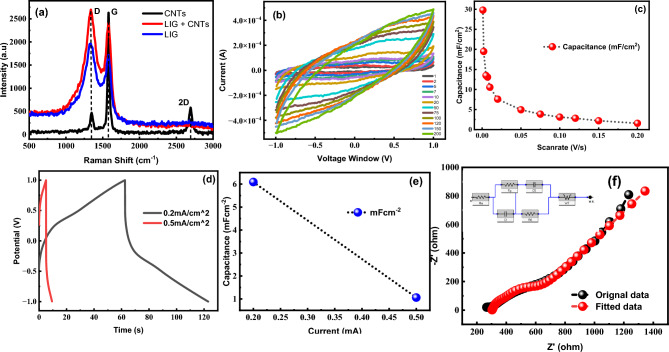
(**a**) Raman spectra of lig, multi-walled carbon nano-tubes (MWCNTs), and LIG coated with MWCNTs, (**b**) Cyclic voltammetry was performed on a LIG supercapacitor using a PVA/H_2_SO_4_ gel electrolyte at a scan rate of 1–200 mV/s and a potential window of − 1 to + 1 V, (**c**) behavior of capacitance by varying scan rates, (**d**) charging and discharging curves of the LIG supercapacitor, (**e**) analyzation of the proposed device's capacitance and energy density, (**f**) EIS of original data and fitted model for LIG electrode, showing equivalent circuit and the solution and charge transfer resistance with overall impedance.

Raman Spectroscopy is an excellent method to characterize carbon nanomaterials. It provides details about the bands and the hybridization of the carbon structure. Figure 5a shows the Raman spectrum of the fabricated film with several lines: D (1326 cm^−1^), G (1579 cm^−1^), and G′ (2690 cm^−1^). The D band indicates defects in the carbon structure, whereas the G band represents the *sp*^2^ hybridized carbon atoms. The G′ band is related to the two-phonon scattering process in graphitic materials. The higher D band in LIG and LIG-MWCNTs indicates the breaking of *sp*^2^ bonds and the formation of more *sp*^3^ bonds Fig. 5a. This change in the hybridization of the carbon structure is likely due to the laser treatment used to fabricate the LIG and LIG-MWCNTs^38^. However, a D band may also be present for several additional causes. Line G′ is often referred to as the "2D peak" and is related to the second-order Raman scattering by Brillouin zone border phonons. The hexagonal structure of *sp*^2^ carbon atoms has defects that cause the first scattering line to appear. Line G, on the other hand, is related to the longitudinal oscillation mode of the carbon atoms.

At scan speeds ranging from 1 to 200 mV/s, cyclic voltammetry was conducted over a voltage window of − 1 to + 1 V. From charge/discharge curves, Eq. (2)^37,38,48^ was used to determine the specific areal capacitance of SC:

2 $$ C_{device} = \frac{I}{{\frac{dv}{{dt}}}} $$

In Eq. (2), I stands for average discharge current and dv/dt for slope of Galvano static discharge curves. The following Eq. (3), is used to compute the areal capacitance:

3 $$ C = \frac{1}{{2 \times s \times (v_{f - } v_{i) } \times \frac{dv}{{dt}}}}\int\limits_{{v_{i} }}^{{v_{f} }} I \left( V \right)dv $$

where s is the electrodes' specific area (1 cm^2^), *v*_*f*_ and *v*_*i*_ are the final and starting voltages (in Volts), and dv/dt is the scan rate (in mV/s). C is the areal capacitance of the LIG and is measured in mF cm^2^. The Eq. (4) and (5) expressed below are used to obtain the real power density ${P}_{A}$ (µW cm^−2^) and the specific areal energy density ${E}_{A}$ (µWh cm^−2^)^48^.

4 $$ E_{A} = \frac{1}{2} \times C \times \left( {\Delta V} \right)^{2} \times \frac{1}{3,600} $$

5 $$ P \, = \frac{{{\varvec{E}}_{{\varvec{A}}} }}{{\varvec{t}}} \times 3,600 $$

PVA/H_2_SO_4_ gel electrolyte was used to examine the electrochemical characteristics of the developed electrodes in two electrode configurations^49^. Ionic gel electrolyte can provide a broader voltage window, enhancing the supercapacitor device's total energy density. Thus, a voltage window of − 1 to 1 V was used to test the performance of the fabricated SC device.

In Fig. 5b, a symmetric fish-type CV curve from the CV analysis indicates that charge is stored through double-layer formation. An ideal CV curve for EDLCs is a perfect square; however, the CV curves for LIG deviate from this trend due to defects and surface roughness. In Fig. 5c, the specific areal capacitance of LIG is shown as a function of the scan rate. At a scan rate of 1 mV/s, the LIG electrode exhibited a high capacitance value of 30 mF/cm^2^, indicating its high energy storage capacity. Moreover, the absence of any significant change in the CV curve with increasing scan rate indicates that the electrode material has good electrochemical performance and rapid electrolyte diffusion^42^. This is important for the practical application of supercapacitor devices, as it indicates that the device can be charged and discharged quickly without significant loss of energy storage capacity. The large channels in the carbon electrode allow transient mass diffusion at higher scan rates while enabling its operation at a high scan rate^47^. With the increasing scan rate, a decreasing trend in capacitance was recorded. For example, capacitances of 19.47, 4.93, and 3.09 mF cm^−2^ were obtained at scan rates of 2, 50, and 100 mV/s, respectively (Fig. 5b). Galvanostatic charge/discharge curves are commonly used to characterize the electrochemical behavior of energy storage devices, such as supercapacitors, by measuring the amount of charge that can be stored and released over time. Galvanostatic charge/discharge curves were obtained to better understand the charge storage process of the electrode. Galvanostatic charge/discharge curves for the LIG electrode displayed a symmetric pattern, as shown in Fig. 5d. This behavior further supports the theory of charge storage by double layer; however, the slight deviation can be attributed to the behavior of the electrolyte. Comparing the charging and discharging times from the GCD curves reveals a discrepancy between the predicted capacitance obtained from the GCD and CV curves. Specifically, the GCD curve yielded a capacitance of 6.09 mF cm^−2^ at a current density of 0.2 mA/cm^2^, as illustrated in Fig. 5e. As shown in , EIS was used to analyze the impedance of the LIG electrode. Due to its insulating properties, the polyimide (PI) sheet exhibits a significantly high overall resistance. The insulating polyimide was converted into a conductive channel using CO_2_ laser ablation. The experimental data of the LIG electrode is shown by the black line in Fig. 5f, while the red line represents a fitted model using an equivalent circuit.

**Figure 6:**
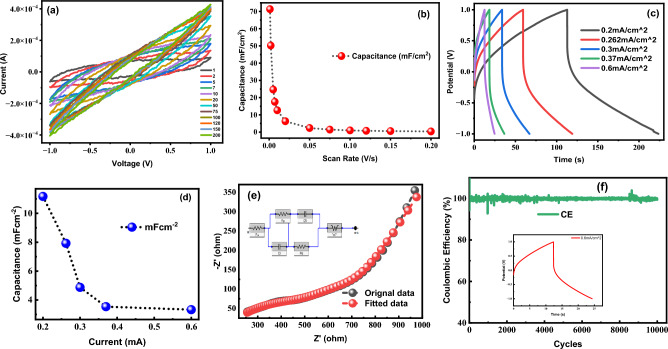
(**a**) Cyclic voltammetry was performed on a LIG-2mg MWCNT coated supercapacitor using a PVA/H_2_SO_4_ gel electrolyte at a scan rate of 1–200 mV/s and a potential window of − 1 to + 1 V. (**b**) Behavior of capacitance by varying scan rates, (**c**) charging and discharging curves of the LIG-2mgMWCNTs supercapacitor, (**d**) analyzation of the proposed device's capacitance and energy density, (**e**) EIS of actual and fitted model for LIG-2mg (MWCNTs) electrode, showing equivalent circuit and charge transfer resistance and overall impedance of the coated sample (MWCNTs), (**f**) stability test of LIG-2mgMWCNT electrode showing the coulombic efficiency at 0.6 mA cm^−2^ current for 10,000 cycles.

After the LIG electrode was designed, we carried out the electrode fabrication process utilizing different MWCNT concentrations to understand the effect of various MWCNT coating concentrations on the properties of LIG electrodes. Specifically, we spray-coated LIG with 2 mg and 5 mg MWCNTs and then analyzed the resulting electrodes to optimize their design. The cyclic voltammetry curves for the LIG/2%CNTs sample are shown in Fig. 6a. An increase in capacitance from 30 to 71.313 mF cm^−2^ at a scan rate of 1 mV/s was recorded, as shown in Fig. 6b. The trend indicates that charge is stored through double-layer formation, with a slight deviation from the ideal case, which could be due to factors such as the presence of impurities or imperfections in the electrode surface. The capacitance of 50.19, 2.36, and 0.993 mF cm^−2^ were obtained at scan rates of 2, 50, and 100 mV/s, respectively, as shown in Fig. 6b. The Galvanostatic charge/discharge curves were obtained to better understand the charge storage process. The symmetric charge/discharge curves of the LIG-coated MWCNTs are shown in Fig. 6c. The electrolyte may cause the minor deviation from the symmetric behavior. These curves suggest that the charge storage process is dominated by the formation of a double layer, which is consistent with the CV data. There is a slight difference between the anticipated capacitance values obtained from the GCD and CV curves. Specifically, the GCD curve yielded a capacitance of 11.17 mF cm^−2^ at a current density of 0.2 mA cm^−2^ (Fig. 6d), whereas the CV data suggested a capacitance of 71.313 mF cm^−2^ at a scan rate of 1 mV/s.

The electrochemical behavior of materials, as elucidated by current–voltage (CV) curves and current density, provides valuable insights into capacitive performance. However, this behavior is multi-faceted and driven by a combination of factors, contributing to the enhanced areal capacitance observed in LIG/2%CNT compared to bare LIG, despite the seemingly modest increase in the CV curve and current density. Several key factors may underpin this enhancement. Firstly, the addition of carbon nanotubes (CNTs) augments charge storage capacity, thanks to their high surface area and superior conductivity, thereby yielding a higher areal capacitance. Secondly, CNTs introduce pseudo-capacitance effects via faradaic redox reactions, further bolstering the composite material's capacitance. Additionally, CNTs enhance electrode conductivity, facilitating swifter charge–discharge rates. Synergistic interactions between LIG and CNTs amplify charge storage. Moreover, the LIG/2%CNT composite may exhibit extended long-term stability, ensuring sustained capacitance over numerous charge–discharge cycles. Lastly, the compatibility of the specific electrolyte utilized can significantly influence capacitive performance, accentuating capacitance despite the incremental CV curve. These multifaceted elements collectively contribute to the comprehensive understanding of capacitive behavior^50–53^.

The study reveals that capacitance can decrease when the coating is applied in excess of the surface pore size. This is because the excess coating will block the pores, and the surface area available for charge storage will be reduced. Additionally, the charging and discharging times from the GCD curves suggest that the device has a moderate coulombic efficiency. The GCD curves suggest that charge accumulation or storage is through the double-layer formation. The capacitance values computed using Galvanostatic charging and discharging curves and those derived using CV analysis differ. Equations (4) and (5) are used to compute the relative power and energy densities. The energy density of the LIG/2%CNTs based SC device is measured as 6.5 μWh cm^−2^ at a corresponding power density of 0.219 mW cm^−2^ which is relatively higher than what is mainly reported^42^ in the literature^40^. This suggests that the device has a high energy storage capacity per unit area. LIG/2%CNTs based electrodes with PVA/H_2_SO_4_ gel electrolyte improve the ionic conductivity of the SC device and increase its capacitance by facilitating the movement of ions. One factor contributing to the observed increase in capacitance may be the porous structure of the MWCNT-coated LIG, which has the potential to provide a larger surface area for the electrolyte, thus facilitating electric double layer formation. A larger surface area and an abundance of wrinkles make it easier for the electrolytes to diffuse and cause electric double layer capacitive diffusion, as reflected by data shown in Fig. 6c,d.

**Figure 7:**
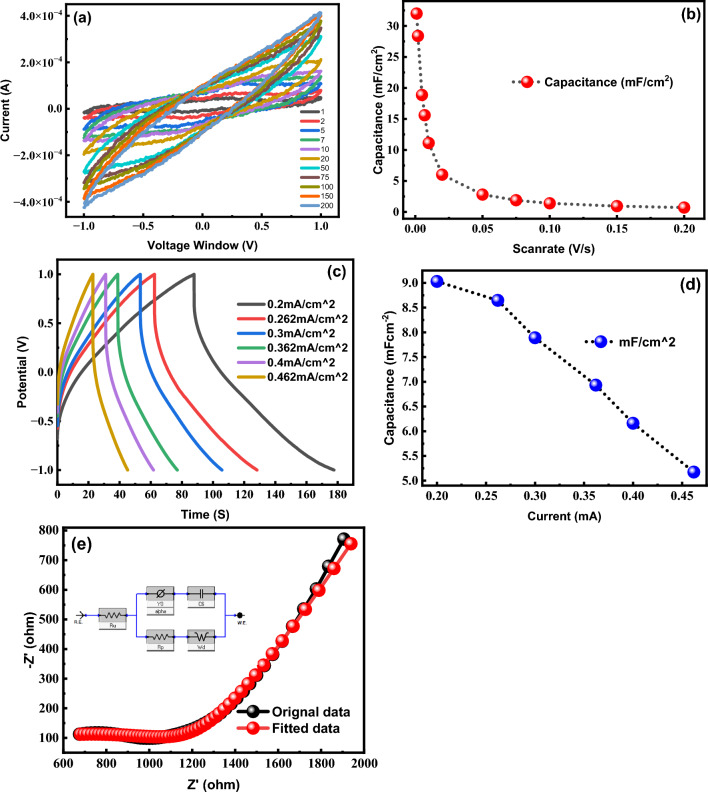
(**a**) Cyclic voltammetry was performed on a LIG-5mg MWCNT coated supercapacitor using a PVA/H_2_SO_4_ gel electrolyte at a scan rate of 1–200 mV/s and a potential window of − 1 to + 1 V, (**b**) behavior of capacitance by varying scan rates, (**c**) charging and discharging curves of the LIG-5mgMWCNTs supercapacitor, (**d**) analyzation of the proposed device's capacitance and energy density, (**e**) EIS of actual and fitted model for LIG-5 mg (MWCNTs) electrode and charge transfer resistance and overall impedance of the coated sample.

EIS was utilized, as depicted in Figs. 6e and 7e, to investigate the LIG electrode that was coated with MWCNT at concentrations of 2 mg and 5 mg respectively. CO_2_ lasers are helpful in carbonizing carbon-based raw materials because of their operating wavelength within the medium- and far-infrared region of the electromagnetic spectrum, where most substrates exhibit strong absorptions. This facilitates rapid and efficient carbonization of the raw materials. Figure 5f demonstrate an increase in impedance following MWCNT coating. In addition, the impedance factor has increased with the increasing concentration of MWCNTs, which results in a pronounced hump in the EIS plot, showing a significant increase in the electrode impedance. To optimize the performance of the cell, it is crucial to minimize the impedance factor, which facilitates the efficient flow of electrolyte ions and electrode interactions, leading to a higher capacitance of the overall cell. The EIS plot in Fig. 5f depicts the actual model by the black line, while the red line represents a fitted model generated using an equivalent circuit. The charge transfer resistance (Rct), internal series resistance (Rs) at high frequencies and Warburg impedance (W) at low frequencies is calculated for all samples and showed in Table 1. Charge transfer resistance is found to be lowest for LIG/2%CNTs sample and have a value of 150 Ω which favors the high capacitive behavious of our electrode. Warburg impedance was also found to be lowest for LIG/2%CNTs favoring good electrical transport which helps in faster ion transport and good electrochemical performance of the device^54,55^_ENREF_47.

**Table 1:** Electrochemical Impedance spectroscopy (EIS) calculated values of Rs, Rct and Warburg impedance for LIG, LIG/2%CNT and LIG/5%CNT electrodes.

Sample No.	R_s_ (Ω)	R_ct_(Ω)	W (Ω)	C_dl_ (mF)
LIG	102.2	1.032 × 10^3^	2.578 × 10^–3^	2.9
LIG/2%CNT	301.5	150	1.2 × 10^–3^	101
LIG/5%CNT	152.2	1.433 × 10^3^	1.282 × 10^–3^	0.342

Figure 7a depicts the cyclic voltammetry curves for the LIG/5%CNTs sample. We obtain a capacitance of 32 mF cm^−2^ at a scan rate of 1 mV/s. Figure 7b illustrate the capacitance measured at scan rates of 2, 50, and 100 mV/s, respectively. We obtained capacitances of 28, 2.7, and 1.39 mF cm^−2^ at scan rates of 2, 50, and 100 mV/s, respectively. These results suggest that the capacitance decreases as the scan rate increases, which is consistent with the behavior of a double-layer capacitor. The symmetric charge/discharge curves shown in Fig. 7c of the LIG-coated MWCNTs suggest that the formation of a double layer still dominates the charge storage mechanism, and any deviations from the ideal behavior are likely due to the electrolyte. However, there is a slight discrepancy between the anticipated capacitance values obtained from the GCD and CV curves. As shown in Fig. 7c, the GCD curve offered a capacitance of 11.17 mF/cm^2^ at a current density of 0.2 mA cm^−2^. Figures 6b and 7b illustrate that the addition of minute amounts of MWCNTs to LIG can significantly enhance the electrical characteristics, capacitance, and energy density of the resulting electrode material. The capacitance of a supercapacitor can be increased by coating it with MWCNTs with optimized pore size. Fabricated electrode LIG/2%CNTs exhibited 71.313 mF cm^−2^ from CV curves at a scan rate of 1 mV/s and 11.17 Mf cm^−2^ from GCD at a current density of 0.2 Ma cm^−2^ as illustrated in Fig. 6a,d. As shown in Fig. 7a, the capacitance of the LIG/5%CNTs coated electrode is lower than that of the LIG coated with 2 mg MWCNTs, but it is still higher than that of the simple LIG electrode. The LIG/5%CNTs exhibits a capacitance of 30 mF cm^−2^ at a scan rate of 1 mV/s and a capacitance of 9.03 mF cm^−2^ at a current density of 0.2 mA cm^−2^ as shown in Fig. 7b,d.

Supercapacitors are becoming increasingly popular due to their high durability and fast charging capabilities. By carefully controlling the pore size and coating the electrode with MWCNTs, the capacitance of the supercapacitor can be significantly increased. Our experimental results, obtained from galvanostatic charge–discharge (GCD) and cyclic voltammetry (CV) measurements at a current density of 0.2 mA/cm^2^ and a scan rate of 2 mV/s, show a capacitance of 11.17 and 51 mF/cm^2^, respectively. These values are higher than those reported in previous studies^27,28,43^, and are summarized in Table 2. Coating multi-walled carbon nanotubes (MWCNTs) on electrodes reduces the charge transfer resistance between the electrolyte ions and the electrode, resulting in an enhanced energy density. Specifically, the measured energy density of MWCNT-coated electrodes was found to be 6.5 µWh/cm^2^, which is significantly higher than that of non-coated electrodes. Additionally, even small amounts of MWCNTs mixed into normally insulating materials can confer significant conductivity, making them attractive for use in the development of lightweight, high-strength components. Such components have potential applications in the field of smart electronics, including portable and wearable devices. The stability test Fig. 6f was conducted at a current density of 0.6 mA cm^−2^, and this time it yielded stable and reliable outcomes. The electrode displayed an impressive 99% coulombic efficiency, which signifies the ratio of charges removed from the supercapacitor relative to the charge used for restoring its original capacity.

**Table 2:** Comparative analysis of the fabricated electrode with varying concentrations of MWCNT.

Sample	MWCNTs coating % on LIG electrode	Capacitance From CV at scan rate of 2 mV/s	Capacitance from GCD	Current density	Energy density	Power density
#	mg	mF cm^−2^	mF cm^−2^	mF cm^−2^	μWh c ${\mathrm{m}}^{-2}$	mW c${\mathrm{m}}^{-2}$
1^st^	LIG-0g	19.47	6.09	0.2	3.38	0.199
2^nd^	LIG-2	51.975	11.17	0.2	6.5	0.219
3^rd^	LIG-5	28.375	9.03	0.2	5.01	0.199

## Conclusion

Efficient energy storage technologies are necessary for rapidly expanding and shifting renewable energy sources. Supercapacitors are one of the widely investigated devices for fast and efficient energy storage. We have fabricated a low-cost, high-performance supercapacitor (SC) with high mechanical robustness and flexibility using laser scribing of a 200 µm thick polyimide sheet. The comb-electrode interspacing is critical for the electrical performance of the capacitor, and it was optimized to 0.6 mm through repeated cyclic voltammetry for different designs. The optimized design was used for the fabrication of the SCs. The fabricated LIG electrode was analyzed using AFM, which showed a surface roughness of 2.03 µm. MWCNTs were spray-coated over the LIG film to enhance the electrochemical properties, conductivity, stability, and flexibility owing to their high capacitive nature, flexibility, and large surface area due to porosity. The LIG/2%CNTs flexible SC exhibits a high specific capacitance of ∼51.9 mF cm^−2^, high energy density of ∼ 6.5 µWh cm^−2^, and a power density of ∼ 0.219 mW cm^−2^. Raman spectroscopy was used for optical analysis, revealing several D and G bands (1362 cm^−1^ and 1579 cm^−1^). The results demonstrate the potential of LIG-MWCNT coated SCs as an effective and versatile energy storage solution for portable and wearable electronics due to their simple fabrication process, low cost, flexibility, and high electrical performance. The fabricated supercapacitor is suitable for powering portable and lightweight consumer devices.

## Data Availability

The datasets used and/or analyzed during the current study are available from the corresponding author on reasonable request.
